# Response to “On the risk of using raw regional data on new HIV infections in France” by Tassi et al.

**DOI:** 10.1002/jia2.26207

**Published:** 2024-01-09

**Authors:** Haoyi Wang, Jean‐Michel Molina, Rosemary Dray‐Spira, Axel J. Schmidt, Ford Hickson, David van de Vijver, Kai J. Jonas

**Affiliations:** ^1^ Department of Work and Social Psychology Maastricht University Maastricht the Netherlands; ^2^ Viroscience Department Erasmus Medical Centre Rotterdam the Netherlands; ^3^ Department of Infectious Diseases, Hôpital Saint‐Louis University of Paris Cité Paris France; ^4^ EPI‐PHARE French National Agency for Medicines and Health Products Safety (ANSM) and French National Health Insurance (CNAM) Saint‐Denis France; ^5^ Sigma Research London School of Hygiene and Tropical Medicine London UK

1

Our colleagues Tassi et al. submitted a Letter to the Editor regarding our recent paper on the spatio‐temporal changes in pre‐exposure prophylaxis (PrEP) uptake among men who have sex with men (MSM) in France [1]. We appreciate their interest and constructive feedback, and we seek to address their concerns and provide further clarification.

Tassi et al.’s primary concern centred around our utilization of unadjusted regional data on new HIV infections notifications, alongside the French subset of EMIS‐2017 survey [2] to estimate the regional HIV‐negative MSM population. While we acknowledge the difference between the unadjusted and adjusted notification data, which considers underreporting and late diagnosis, our approach relies on the number of newly diagnosed infections (including both recent and late diagnoses), rather than data on new infections.

In our paper, we modelled the regional risk of the newly diagnosed HIV in 2017 in France through a Bayesian spatial analysis using self‐reported data from EMIS‐2017. However, unlike surveillance notification data, self‐reported survey‐based data cannot be adjusted for underreporting or late diagnosis. To ensure high homogeneity in data sources, we prioritized consistency over potentially higher data quality. Unfortunately, there remains a trade‐off either way. We acknowledge this limitation and caution the interpretation of our model's results as potential underestimations, as emphasized in the Limitations in our paper.

We acknowledge our estimates based on unadjusted data should be interpreted with caution, especially considering potential heterogenous underreporting rates among regions, notably observed in Centre‐Val‐de‐Loire (CVL). Given the limited published data at the same level of detail, this is a limitation we must accept. However, we believe that this limitation, which could apply to any region, is unlikely to fully explain the result that is the subject of criticism, especially on the MSM proportion among adult men in CVL being half that of other French regions, as how Tassi et al. suggested. To delve deeper, we further explored the local MSM population concentration factor (K) within CVL using a previously validated approach [3]. Among the six CVL départements, four (except for Indre‐et‐Loire [K = 0.9] and Loiret [K = 0.8]) demonstrated particularly low MSM concentrations (K ranged from 0.4 to 0.5), meaning that while the first two départements are close to the French average (K = 1.0), the other four départements have only half or less MSM as expected from the national average. These findings, based on EMIS‐2017 data, affirm a notably lower concentration of MSM in the CVL region [1].

Regarding using Bayesian inference, it is important to clarify that our approach did not directly apply it to estimate the regional HIV‐negative MSM population. Instead, we employed Bayesian spatial analysis as an indirect approach to model the regional risk of newly diagnosed HIV, based on EMIS‐2017 data, as a spatial proxy, assuming that our model closely approximated reality [4]. Consequently, our indirect approach did not necessitate direct prior knowledge of the regional MSM population size, rendering it irrelevant to our approach.

We appreciate Tassi et al.’s suggestion to leverage clinical data warehouses and healthcare insurance data for future research. We share their belief in the potential of detailed clinical and insurance data to improve raw surveillance notification data quality. Consequently, we recommend a replication study of our modelling approach once national clinical data warehouses are established. Such an effort would likely yield a more precise estimate of the regional HIV‐negative MSM population, offering broader implications for public health.

We welcome the critical insights provided by Tassi et al. and hope that this response addresses their concerns. We remain committed to advancing our understanding of HIV epidemiology and its impact on vulnerable populations.

## COMPETING INTERESTS

The authors declare no competing interests.

## AUTHORS’ CONTRIBUTIONS

HW, DVDV and KJJ conceptualized this research; HW, JM‐M, RD‐S, AJS, FH and KJJ collected the data for this research; HW analysed the data; HW and KJJ drafted the manuscript; all authors critically revised the manuscript for intellectual content; AJS and FH edited the manuscript. All authors read and approved the final version of the manuscript.

## Data Availability

Data sharing not applicable to this article as no datasets were generated or analysed during the current study.
